# Supplementary material for: Fever-like temperature impacts on Staphylococcus aureus and Pseudomonas aeruginosa interaction, physiology, and virulence both in vitro and in vivo
Source: BMC Biol. 2024 Feb 5;22:27. doi: 10.1186/s12915-024-01830-3 (PMC10845740; doi:10.1186/s12915-024-01830-3)
Supplement: Supplementary file 1 — Additional file 1: Fig. S1. Pigment production and CFU counting under longer incubation periods. Fig. S2. Differentially expressed genes in S. aureus USA300. Fig. S3. Differentially expressed genes in P. aeruginosa PAO1. Fig. S4. Functional classification for S. aureus differentially expressed genes. Fig. S5. Functional classification for P. aeruginosa differentially expressed genes. Fig. S6. PCA analysis of differentially expressed genes. Fig. S7. Cytochrome expression for S. aureus and P. aeruginosa. Fig. S8. Interaction ANOVA of differentially expressed genes. Fig. S9. Representative images for virulence factor in vitro analysis. [file 12915_2024_1830_MOESM1_ESM.pdf]

A

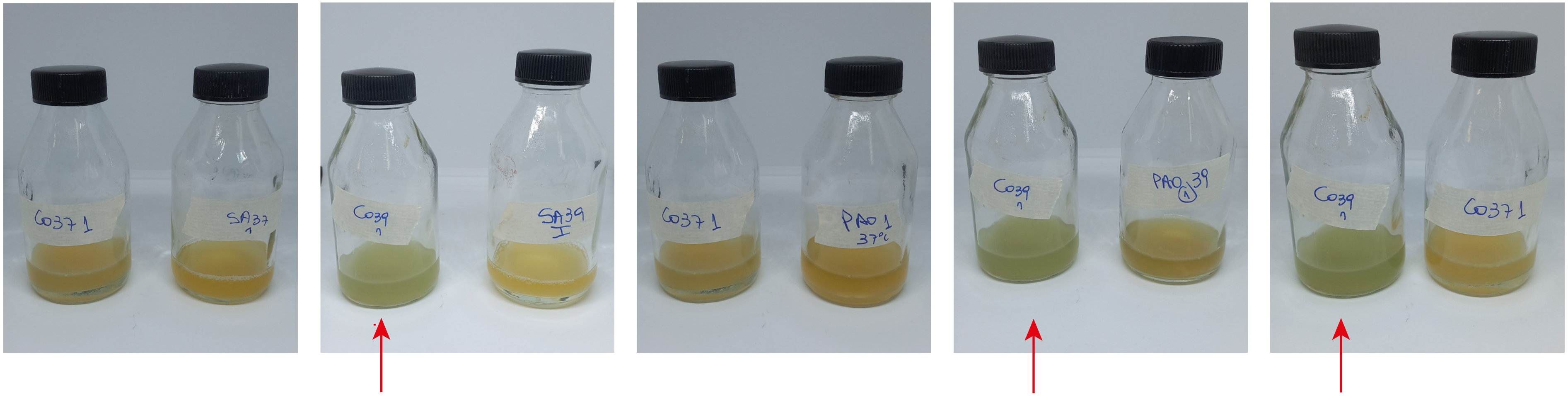

B

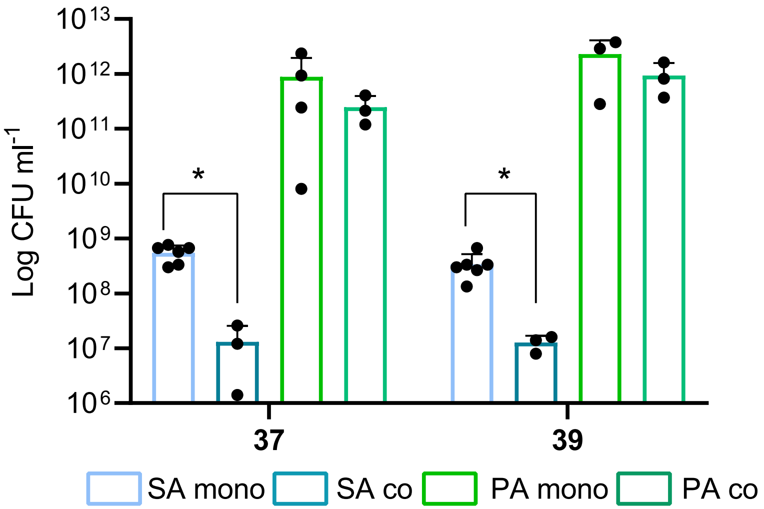

Fig. S1: A. SA, PA or cocultures incubated for 24 h at 37ºC or 39ºC. Pigment production in cocultures at 39ºC is marked with an arrow.
B. SA CFU/ml and PA CFU/ml counting in monocultures and cocultures incubated at 37ºC or 39ºC for 24 h. * denotes significant differences.
Even though data is displayed in the same graph, the following comparisons were performed independently using 1-way Anova:
SA mono 37ºC vs SA co 37ºC P = 0.0008, SA mono 39ºC vs SA co 39ºC P = 0.0311

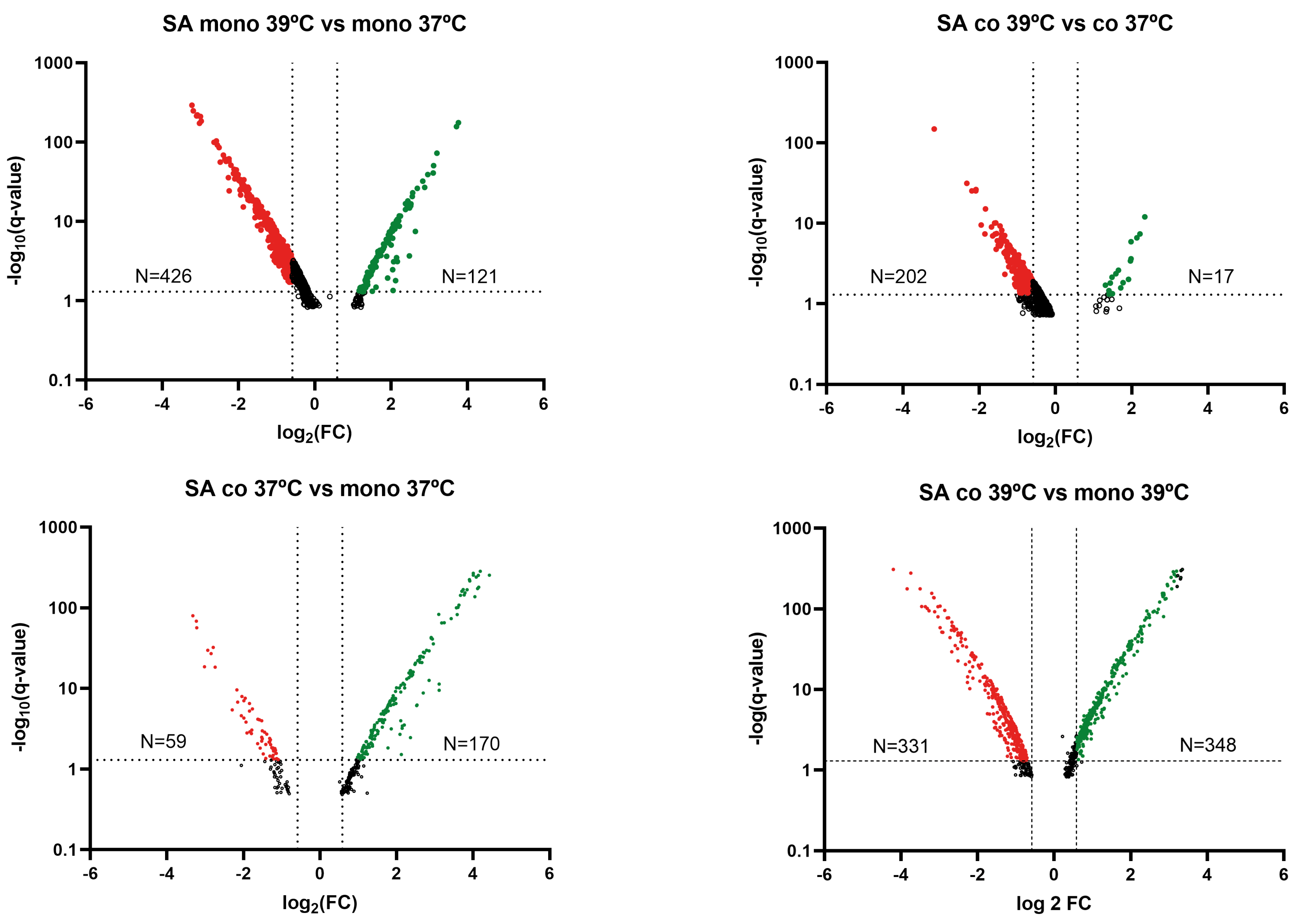

Fig S2: Volcano plots depicting –log10 (Q-value) versus log2 (Fold Change -FC-) for SA genes under different culture conditions (Fig.1). Differentially expressed genes (DEGs) are represented by colored dots. Green dots represent upregulated genes (P-value and Q-value <0.05, Fold change >1.5) and red dots represent downregulated genes (P-value and Q-value <0.05, Fold change < -1.5). For the construction of the Volcano plots, genes were initially filtered based on their P-values. All resulting genes were plotted and filtered again by Q-value.

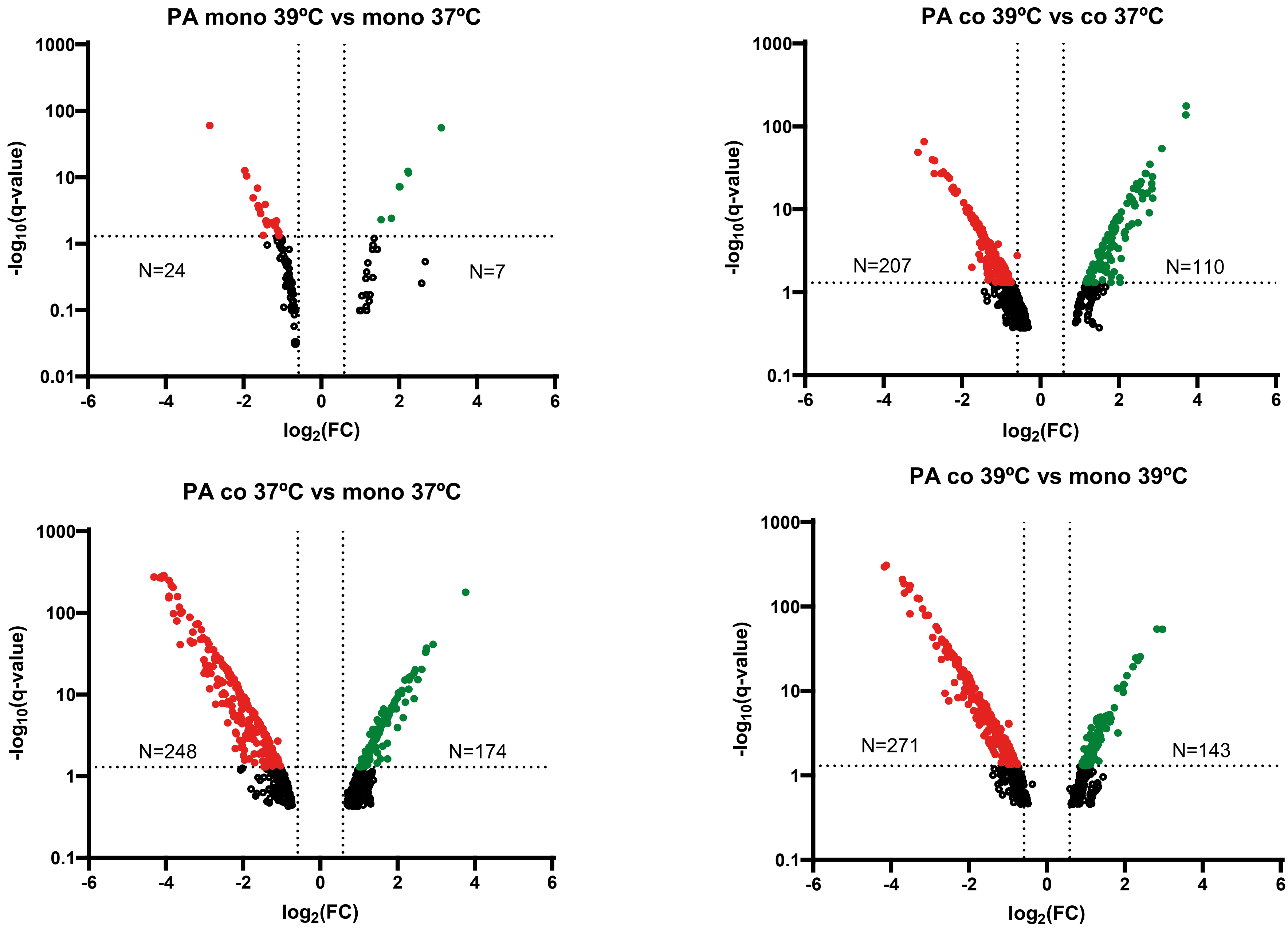

Fig S3: Volcano plots depicting –log10 (Q-value) versus log2 (Fold Change -FC-) for SA genes under different culture conditions (Fig.1). Differentially expressed genes (DEGs) are represented by colored dots. Green dots represent upregulated genes (P-value and Q-value <0.05, Fold change >1.5) and red dots represent downregulated genes (P-value and Q-value <0.05, Fold change < -1.5). For the construction of the Volcano plots, genes were initially filtered based on their P-values. All resulting genes were plotted and filtered again by Q-value.

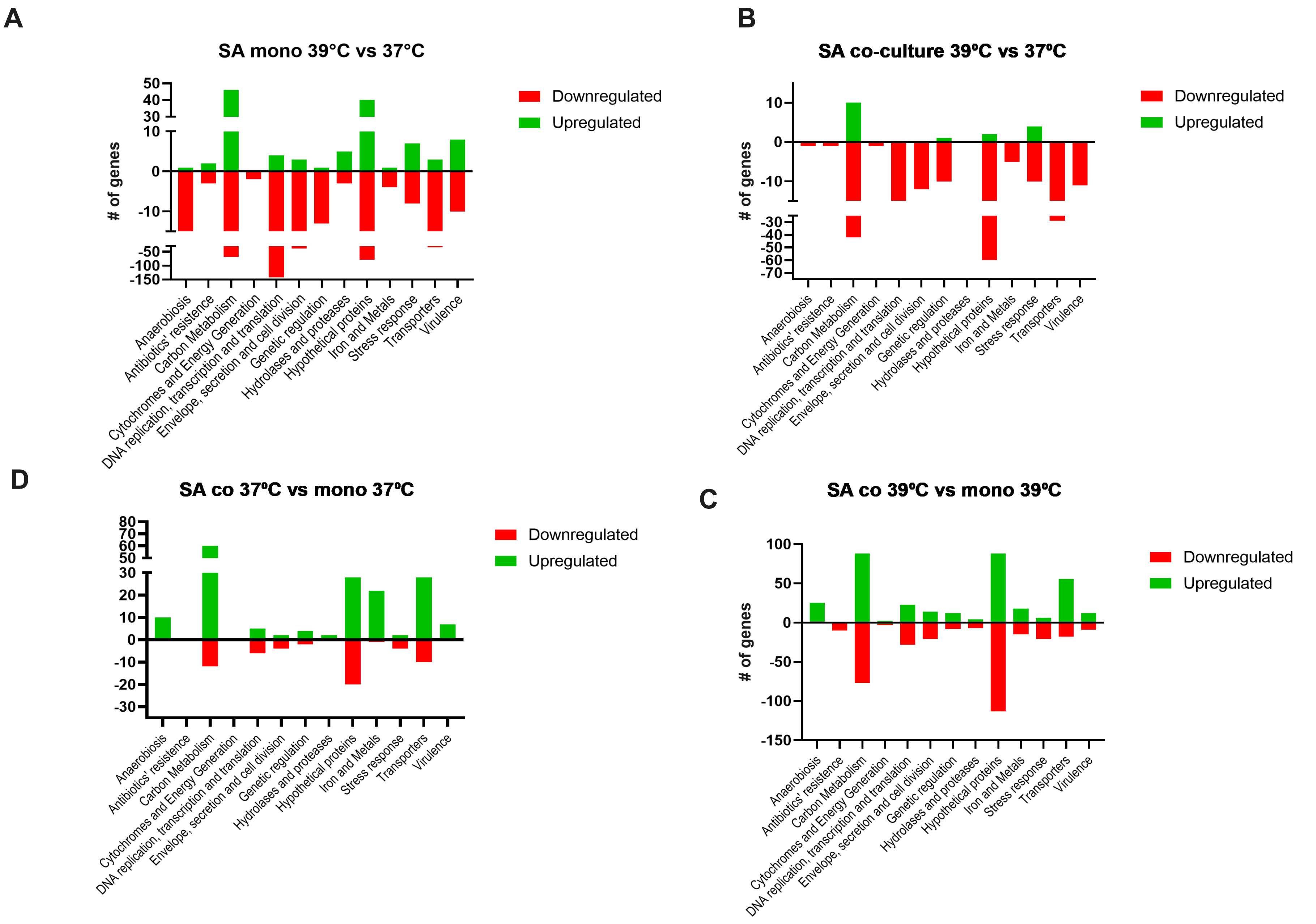

Fig S4: Functional classification for SA differentially expressed genes after incubation under different culture conditions as described in Fig.S1. Upregulated genes are represented in green while downregulated genes are shown in red.

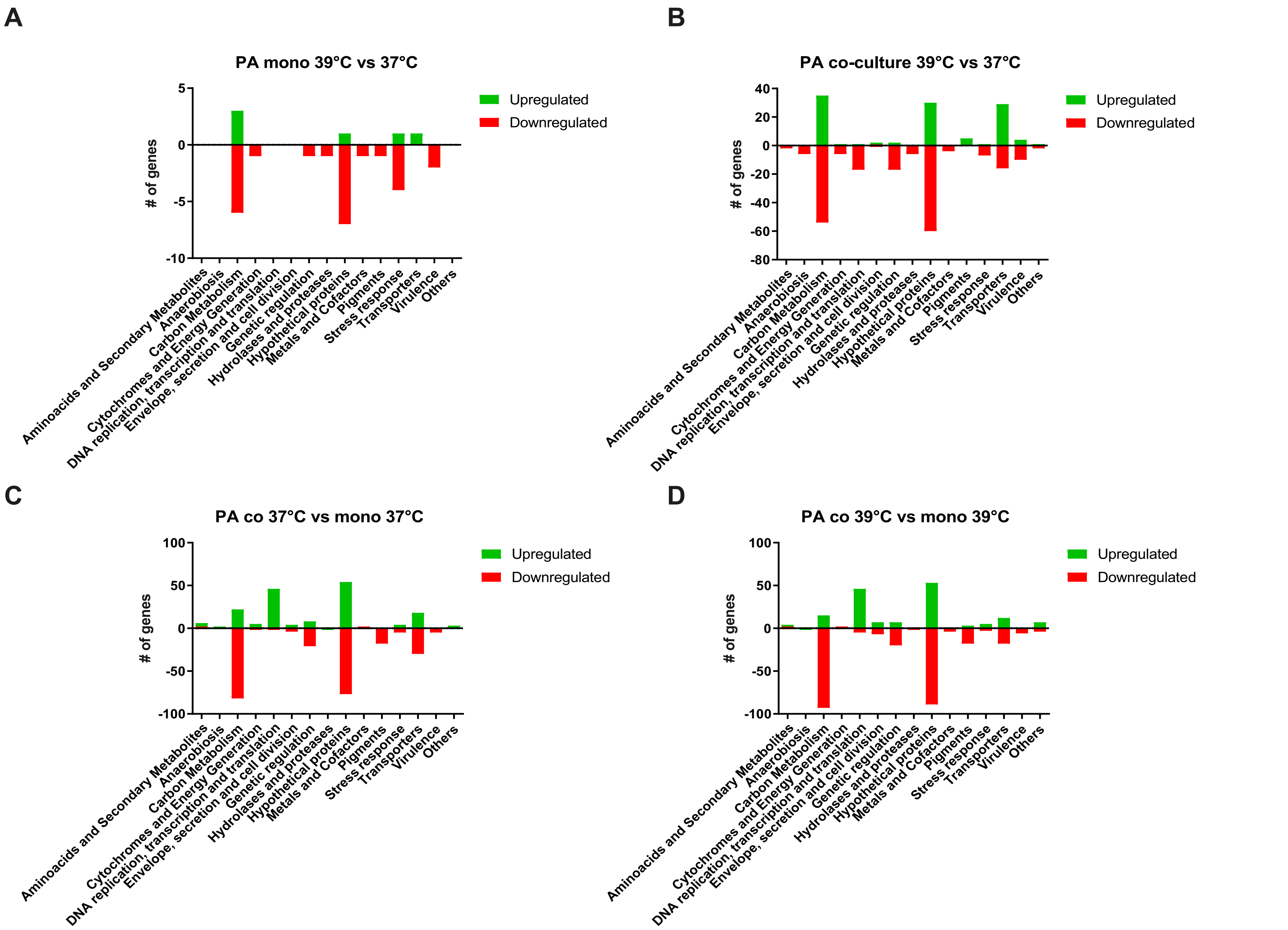

Fig S5: Functional classification for PA differentially expressed genes after incubation under different culture conditions as described in Fig.S1. Upregulated genes are represented in green while downregulated genes are shown in red.

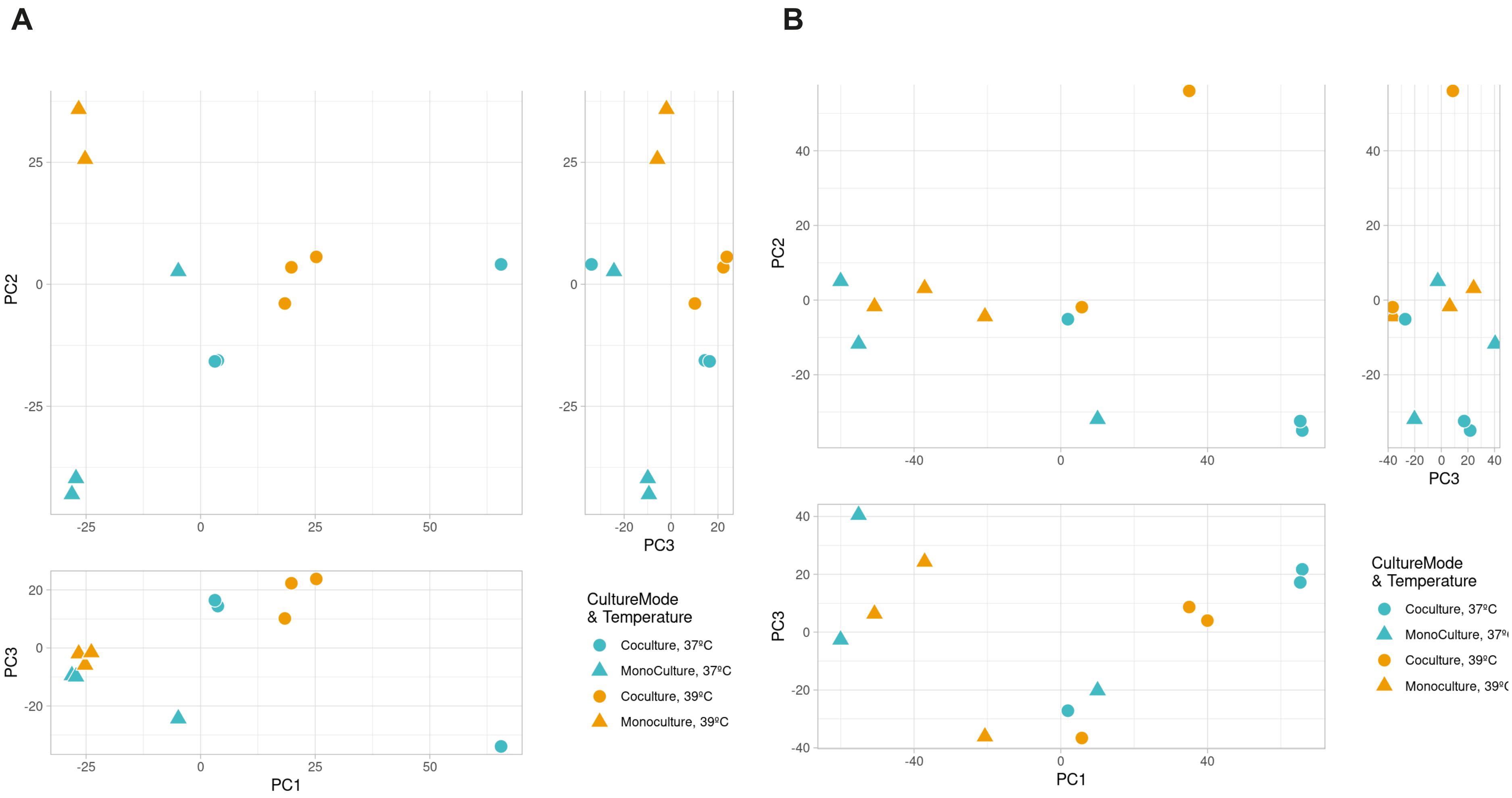

Fig S6: A. Principal Component Analysis for SA expression profile. B. Principal Component Analysis for PA expression profile.

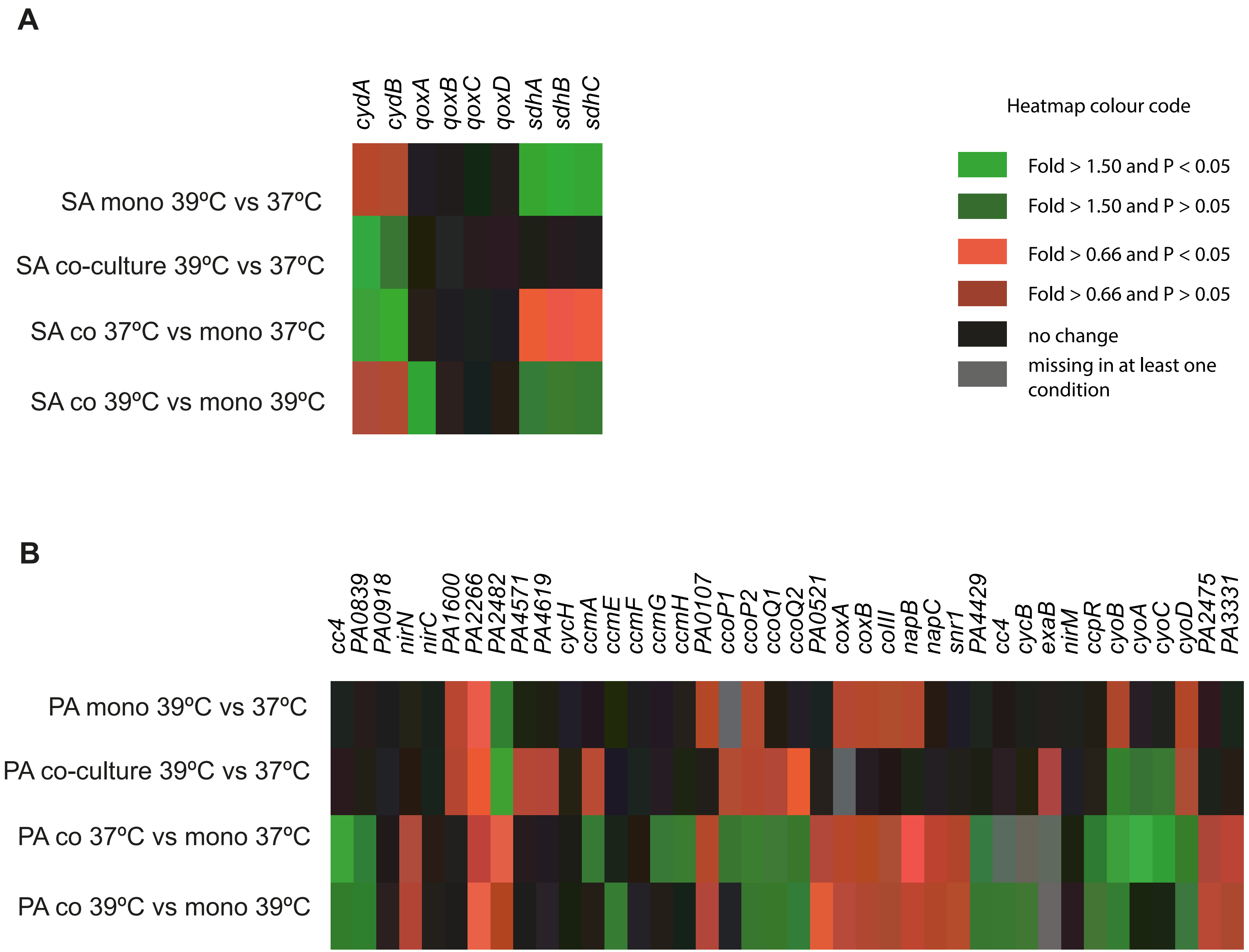

Fig S7: A. Number of differentially expressed genes detected by RNAseq. B. Cytochrome expression for SA. Upregulated genes: dark green. Genes with a fold-change higher than 1.5 but with P>0.05 are colored in light green. Downregulated genes are presented in red. Genes with a fold-change lower than 0.66 P>0.05 are colored in light red. Genes with fold changes between 0.67 and 1.49 are shown in black. Grey indicates that expression of the gene is not detected in one or both conditions. C. Cytochrome expression for PA. Upregulated genes: dark green. Genes with a fold-change higher than 1.5 but with P>0.05 are colored in light green. Dow nregulated genes are presented in red. Genes with a fold-change lower than 0.66 P>0.05 are colored in light red. Genes with fold changes between 0.67 and 1.49 are shown in black. Grey indicates that expression of the gene is not detected in one or both conditions.

A

B

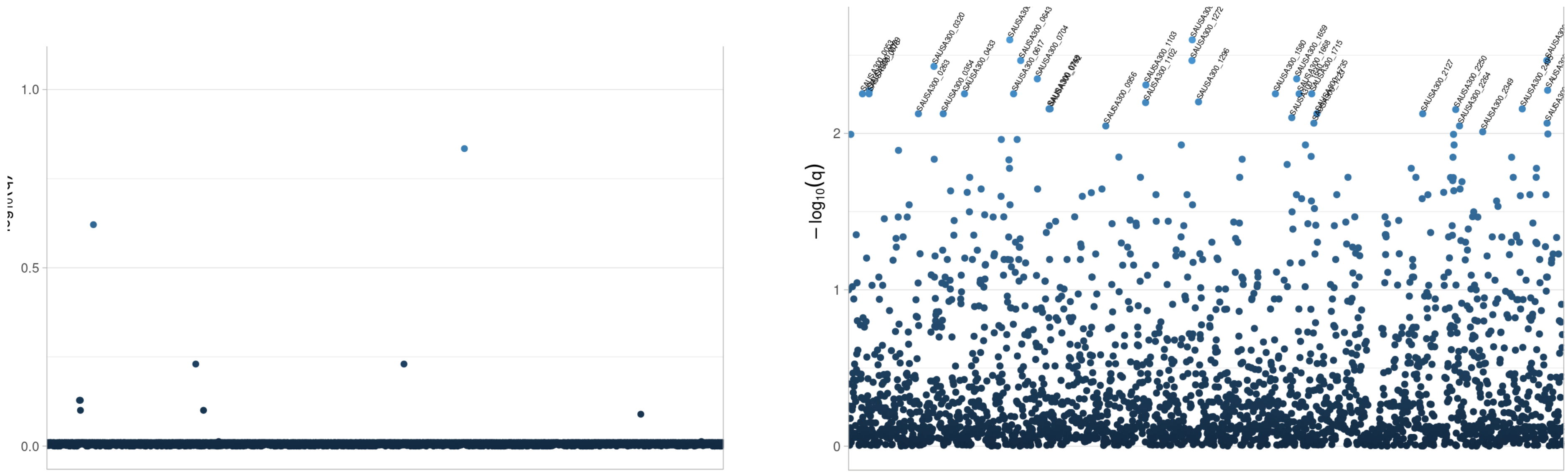

Fig S8: A.Interaction ANOVA for PA expressed genes. Analysis was performed using R language. B. Interaction ANOVA for SA expressed genes

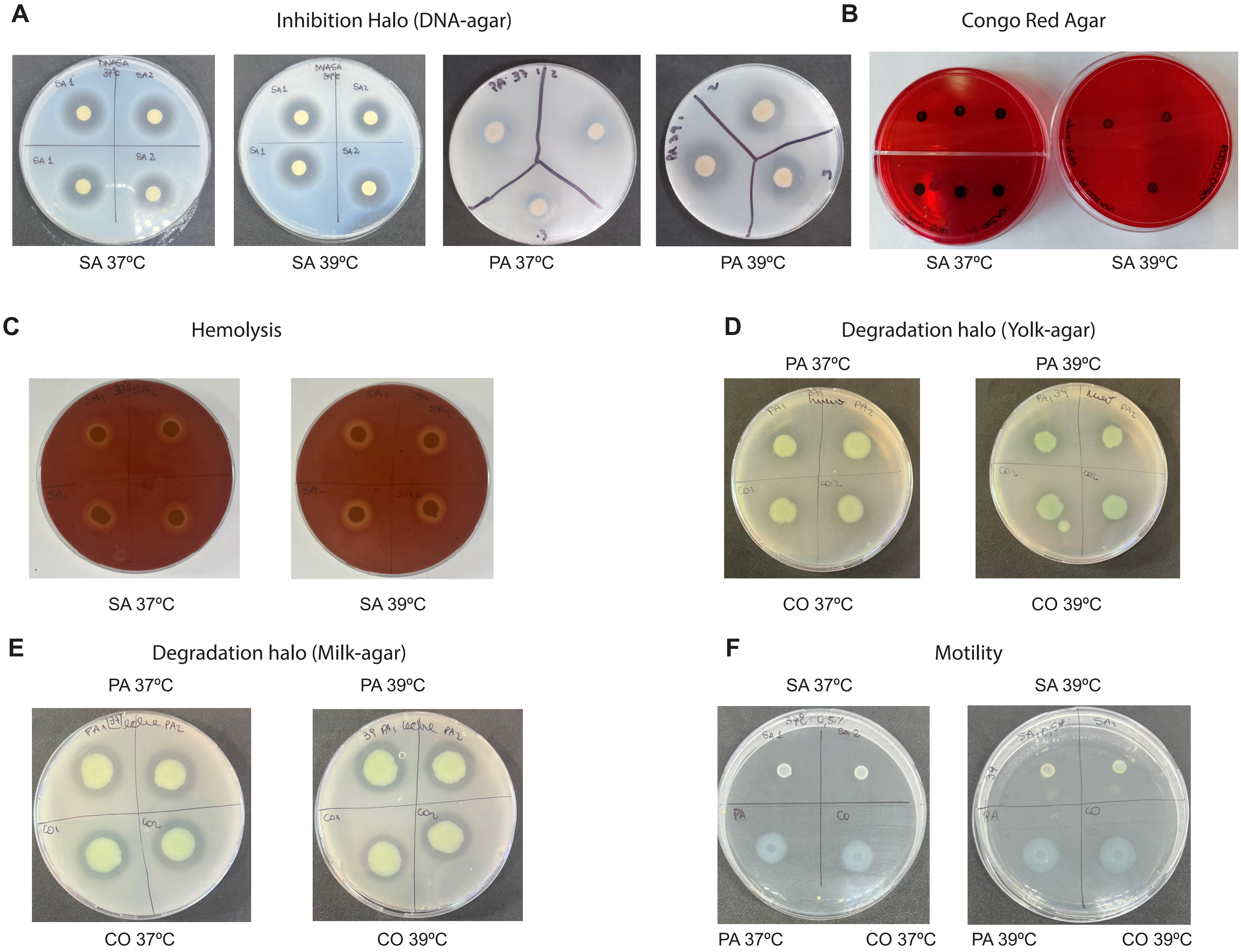

Fig S9: Representative images for virulence factor in vitro analysis. A.Inhibition halo in DNA-agar B. SA colonies in Congo Red Agar C. SA hemolysis halo D. Degradation halo in Yolk-agar E. Degradation halo in Milk-agar F. Motility assays
