# Supplementary material for: Fever-like temperature impacts on Staphylococcus aureus and Pseudomonas aeruginosa interaction, physiology, and virulence both in vitro and in vivo
Source: BMC Biol. 2024 Feb 5;22:27. doi: 10.1186/s12915-024-01830-3 (PMC10845740; doi:10.1186/s12915-024-01830-3)
Supplement: Supplementary file 4 — Additional file 4: Table S3. Gene Ontology Analysis for enriched pathways in S. aureus expression profile. Table S4. Gene Ontology Analysis for enriched pathways in P. aeruginosa PAO1 expression profile. Table S5. Metabolic features of S. aureus. Table S6. Relevant metabolic features in P. aeruginosa. Table S7. genes related to virulence that present in its expression interaction between temperature and culture condition in S. aureus. Table S8. Antibiotic sensitivity for S. aureus. Table S9. Antibiotic sensitivity for P. aeruginosa [file 12915_2024_1830_MOESM4_ESM.pdf]

Table S3: Gene Ontology Analysis for enriched pathways in *S. aureus* expression profile

Enriched GEO pathways			
**Monoculture 39 vs 37**	**Cocultures 39 vs 37**	**Coculture 37 vs monoculture 37**	**Coculture 39 vs monoculture 39**
Urea cycle	Superpathway of tetrahydrofolate biosynthesis and salvage	L-leucine biosynthesis	L-glutamate biosynthesis I
L-arginine degradation VI (arginase 2 pathway)	Tetrahydrofolate salvage from 5,10-methenyltetrahydrofolate	L-isoleucine biosynthesis I (from threonine)	L-isoleucine biosynthesis I (from threonine)
L-histidine degradation I	5-aminoimidazole ribonucleotide biosynthesis	Superpathway of branched chain amino acid biosynthesis	Superpathway of branched chain amino acid biosynthesis
L-arginine biosynthesis II (acetyl cycle)	Inosine-5'-phosphate biosynthesis I	L-glutamate biosynthesis I/III	
L-arginine degradation I (arginase pathway)	UMP biosynthesis I superpathway of pyrimidine deoxyribonucleotides de novo biosynthesis	L-glutamine degradation I/ II	L-glutamine degradation I
Glycine cleavage	Glycine cleavage	L-serine degradation	L-serine degradation
Formate assimilation into 5,10-methylenetetrahydrofolate	Superpathway of pyrimidine ribonucleotides *de novo* biosynthesis	L-asparagine biosynthesis III (tRNA-dependent)	Pyridoxal 5'-phosphate biosynthesis II
Triacylglycerol degradation	Triacylglycerol degradation I	L-alanine degradation IV	L-threonine degradation
Flavin biosynthesis I	Inosine 5'-phosphate degradation	L-homoserine biosynthesis	
2-oxoglutarate decarboxylation to succinyl-CoA	Guanosine nucleotides degradation III	Pyruvate fermentation	Pyruvate fermentation I/V
5-aminoimidazole ribonucleotide biosynthesis I	di-trans poly-cis-undecaprenyl phosphate biosynthesis	ethanol degradation I	Ethanol degradation I/II
Gluconeogenesis I	Mevalonate degradation	Heme degradation VI	Heme degradation VI
Folate polyglutamylation	Molybdenum cofactor biosynthesis	Ammonia assimilation cycle III	Glycerol and glycerophosphodiester degradation
TCA cycle I	Biotin-carboxyl carrier protein assembly	Glutaminyl-tRNA-biosynthesis via transamidation	Heterolactic fermentation
Glycerol degradation V	UTP and CTP dephosphorylation folate transformations III		Pyruvate fermentation to lactate
Mixed acid fermentation	Tetrapyrrole biosynthesis I (from glutamate)		Mixed acid fermentation
Staphyloxanthin biosynthesis	Lipoteichoic acid biosynthesis	staphyloferrin B biosynthesis	Siroheme biosynthesis

Table S4: Gene Ontology Analysis for enriched pathways in *P. aeruginosa* PAO1 expression profile

**Enriched GEO pathways**			
**Monoculture 39 vs 37**	**Cocultures 39 vs 37**	**Coculture 37 vs monoculture 37**	**Coculture 39 vs monoculture 39**
Adenosyl-L-methionine salvage II	Alanine biosynthesis II	Alanine biosynthesis III	Isopropylamine degradation
Mixed acid fermentation	2-oxoisovalerate decarboxylation to isobutanoyl-CoA	ATP biosynthesis	ATP biosynthesis
Glycerol degradation I	Biotin-carboxyl carrier protein assembly	Guanosine ribonucleotides *de novo* biosynthesis	tRNA charging
Glycerophosphodiester degradation	Branched-chain fatty acid biosynthesis	Heme biosynthesis II (oxygen-independent)	L-serine biosynthesis I
L-methionine biosynthesis	Superpathway of acetate utilization and formation	PRPP biosynthesis	L-tryptophan biosynthesis
Reactive oxygen species degradation	Glycine betaine degradation I	Biosynthesis from glutamate	L-phenylalanine biosynthesis
Glyoxylate bypass	Folate polyglutamylation	L-arginine degradation IX	Chorismate biosynthesis I
D-gluconate degradation	Superpathway of L-serine and glycine biosynthesis I	Arginine:pyruvate transaminase pathway	3-dehydroquinate biosynthesis I
Fatty acid biosynthesis initiation (type II)	Pyocyanin biosynthesis	Selenate reduction	Selenate reduction
Thiosulfate oxidation III	2-methylcitrate cycle I		Sulfate activation for sulfonation
	Octane oxidation		Assimilatory sulfate reduction IV
	Stearate biosynthesis II		Lipopolysaccharide biosynthesis
	Glycine biosynthesis I		Gluconeogenesis I
	Palmitate biosynthesis		Polyhydroxydecanoate biosynthesis
	L-tyrosine degradation I		tRNA processing
	L-valine degradation I		Spermidine biosynthesis
			Coenzyme A biosynthesis I
			Lipid A biosynthesis I

<u>Table S5</u>: Metabolic features of *S. aureus*

Metabolic Pathway/Branch	Up-regulated genes	Down-regulated genes	Genes with increased expression (non-significative differences)	Genes with decreased expression (non-significative differences)
**Mono 39 vs 37**				
**Arginine metabolism**	*arg-G, argH, rocD, gudB*		*argJ, rocF, putA*	*argC, argF*
**TCA**	*mqo, gltA, icd, sucA, sucB*		*sucC, sdhA sdhB*	
**Glycolysis**		*plkA, fbaA*		
**Peripheral feeding pathways**	*gudB, hutG*		*hutH, hutI, hutU, rocA putA*	
**Fermentative metabolism**	*fdh*	*ldhA, pflAB, ackA pta, budA, budB*		*gltA, gltB*
**Nitrate reduction**		*narK, narHIJ, nirBD, nirR, nreABC*		
**Staphyloxantin biosynthesis**	*crtN, crtM, crtP, crtQ crtO*			
**Co vs mono 37**				
**L-isoleucine, L-valine and L-leucine**	*ilvA, ilvB , ilvC , ilvD, ilvN, leuA leuB*			
**Fermentative metabolism**	*ldhA, pflA, pflB*			
**Staphyloferrin B biosynthesis**	*sfnaA, sfnaB, sfnaC ,sfnaD*			
**Co vs mono 39**				
**Glycolysis**	*glk, pfka, tpiA, gap, pgk, pgm, pyk*		*pgi, fbaA*	
**TCA**				
**Cytochrome**		*sdhC, sdhA, sdhB*		
**Staphyloferrin B biosynthesis**	*sfnaA, sfnaB, sfnaC, sfnaD*			
**Co 37 vs 39**				
**Fermentative metabolism**		*ldh, budB,*		

Table S6: Relevant metabolic features in *P. aeruginosa*

Metabolic Pathway/Branch or cellular function	Up-regulated genes	Down-regulated genes	Genes with increased expression (non-significative differences)	Genes with decreased expression (non-significative differences)
**Mono 39 vs 37**				
**Periplasmic glucose oxidation**		*kgut, kguk, kgD, kgnt, eda*		
**Co vs mono 37**				
**Peripheral fructose catabolic pathway**		*fruA, fruK fruI*		
**Assimilatory sulfonate reduction**	*cysD, cysN, cysH,* alkane monoxygenase			
**L-lactate oxidation**	*lldD, lldP*			
**Co vs mono 39**				
**Periplasmic glucose oxidation pathway**		*gdc, kgut, kguK, kguD, glk*		
**Glyoxylate shunt**	*icl, sucC, sucD*			
**Lactate oxidation**	*lldD, lldP*			
**Co 39 vs 37**				
**Ethanol oxidation**	*pqqC, pqqD, exaC, erbR*		*pqqA, pqqB, pqqE, erbS*	
**Anaerobic metabolism**		*arcD, nrdD, narK1, narK2, dnr, aer2, hcnB*		

Table S7: genes related to virulence that present in its expression interaction between temperature and culture condition in SA using Multifactorial Anova with Benjamini-Hochberg method.

Locus tag	Gen	Product	Q interaction
SAUSA300_1989	*agrB*	accessory gene regulator protein B	0.0443833
SAUSA300_1991	*agrC*	accessory gene regulator protein C	0.0444769
SAUSA300_1992	*agrA*	accessory gene regulator protein A	0.0458893
SAUSA300_1067	*psmβ1*	anti protein	0.0374364
SAUSA300_1068	*psmβ2*	anti protein	0.0389214
SAUSA300_1988	*hld*	delta-hemolysin	0.0341659

<u>Table S8:</u> Antibiotic sensitivity for *S. aureus* in Sensitrine ARGPF plates incubated at different temperatures.

**Antibiotic**	**MIC (µg/ml)**	
	37°C	39°C
Vancomycin	1	1
Chloramphenicol	8	8
Penicillin	>8	>8
Rifampicin	≤0.5	≤0.5
Ampicillin	>8	>8
Tigecycline	0.25	0.25
**Moxifloxacin**	**2**	**4**
Erythromycin	>4	>4
oxacilin+2%NaCl	>4	>4
Levofloxacin	>4	>4
Nitrofurantoin	≤32	≤32
**Daptomycin**	**≤0.5**	**1**
Linezolid	2	2
Ciprofloxacin	>4	>4
Tetracycline	≤2	≤2
Gentamicin	≤2	≤2
Minocycline	≤4	≤4
Clindamycin	≤0.5	≤0.5
Streptomycin 1000	NEG	NEG
Gentamicin 500	NEG	NEG

<u>Table S9:</u> Antibiotic sensitivity for *P. aeruginosa* PAO1 in Sensitrine ARGNP plates incubated at different temperatures.

**Antibiotic**	**MIC (µg/ml)**	
	37°C	39°C
Ceftazimide	≤2	≤2
Ceftazimide/clavunalic acid	0,5/4	0.5/4
Ceftaximide/clavunalic acid	>2/4	>2/4
**Cefotaxime**	**32**	**8**
Ampicilin/sulbactam (2:1 ratio)	>16/8	>16/8
Ampicillin	8<	8<
Piperacilin/tazobactam constant 4	≤8/4	≤8/4
Cepefime	≤2	≤2
Meropenem	≤1	≤1
Cephalothin	>32	>32
Iminipem	2	2
Amikacin	≤8	≤8
Levofloxacin	4	4
Gentamycin	≤4	≤4
Ciprofloxacin	>2	>2
**Minocycline**	**8**	**>8**
Tigecycline	>2	>2
Cefuroxime	>16	>16
Ertapenem	>2	>2
Colistin	≤1	≤1
Cefoxitin	>16	>16
Doripenem	≤4	≤4
Rifampin	>8	>8
Nitrofurantoin	>64	>64
Fosfomycin+glucose 6 phosphare	>fos+ 64	>fos+ 64
Trimethoprim/sulfamethoxazole	>2/38	>2/38
Chloramphenicol	>16	>16
Amoxicilin/clavulanic acid 2:1 ratio	>16/8	>16/8
Aztreonam	≤8	≤8
Nalidixic acid	>16	>16
